# Efficacy and safety of mesenchymal stem cell therapy in liver cirrhosis: a systematic review and meta-analysis

**DOI:** 10.1186/s13287-023-03518-x

**Published:** 2023-10-20

**Authors:** Wenming Lu, Jiayang Qu, Longxiang Yan, Xingkun Tang, Xuesong Wang, Anqi Ye, Zhengwei Zou, Lincai Li, Junsong Ye, Lin Zhou

**Affiliations:** 1 Subcenter for Stem Cell Clinical Translation, First Affiliated Hospital of Gannan Medical University, Ganzhou, 341000 Jiangxi People’s Republic of China; 2 School of Rehabilitation Medicine, Gannan Medical University, Ganzhou, 341000 Jiangxi People’s Republic of China; 3 Ganzhou Key Laboratory of Stem Cell and Regenerative Medicine, Ganzhou, 341000 Jiangxi People’s Republic of China; 4 The First Clinical College of Gannan Medical University, Ganzhou, 341000 Jiangxi People’s Republic of China; 5 Key Laboratory of Prevention and Treatment of Cardiovascular and Cerebrovascular Diseases, Ministry of Education, Gannan Medical University, Ganzhou, 341000 Jiangxi People’s Republic of China; 6 Key Laboratory of Biomaterials and Biofabrication in Tissue Engineering of Jiangxi Province, Gannan Medical University, Ganzhou, 341000 Jiangxi People’s Republic of China

**Keywords:** Mesenchymal stem cells, Liver cirrhosis, Efficacy, Safety, Meta-analysis

## Abstract

**Aim:**

Although the efficacy and safety of mesenchymal stem cell therapy for liver cirrhosis have been demonstrated in several studies. Clinical cases of mesenchymal stem cell therapy for patients with liver cirrhosis are limited and these studies lack the consistency of treatment effects. This article aimed to systematically investigate the efficacy and safety of mesenchymal stem cells in the treatment of liver cirrhosis.

**Method:**

The data source included PubMed/Medline, Web of Science, EMBASE, and Cochrane Library, from inception to May 2023. Literature was screened by the PICOS principle, followed by literature quality evaluation to assess the risk of bias. Finally, the data from each study's outcome indicators were extracted for a combined analysis. Outcome indicators of the assessment included liver functions and adverse events. Statistical analysis was performed using Review Manager 5.4.

**Results:**

A total of 11 clinical trials met the selection criteria. The pooled analysis' findings demonstrated that both primary and secondary indicators had improved. Compared to the control group, infusion of mesenchymal stem cells significantly increased ALB levels in 2 weeks, 1 month, 3 months, and 6 months, and significantly decreased MELD score in 1 month, 2 months, and 6 months, according to a subgroup analysis using a random-effects model. Additionally, the hepatic arterial injection favored improvements in MELD score and ALB levels. Importantly, none of the included studies indicated any severe adverse effects.

**Conclusion:**

The results showed that mesenchymal stem cell was effective and safe in the treatment of liver cirrhosis, improving liver function (such as a decrease in MELD score and an increase in ALB levels) in patients with liver cirrhosis and exerting protective effects on complications of liver cirrhosis and the incidence of hepatocellular carcinoma. Although the results of the subgroup analysis were informative for the selection of mesenchymal stem cells for clinical treatment, a large number of high-quality randomized controlled trials validations are still needed.

**Supplementary Information:**

The online version contains supplementary material available at 10.1186/s13287-023-03518-x.

## Introduction

Chronic liver disease severely threatens global public health, accounting for about two million mortality rates worldwide annually. Notably, nearly half of the mortalities are liver cirrhosis (LC) patients, and subsequently followed by viral hepatitis and liver cancer patients [1]. LC is developed from chronic liver disease with various etiologies, including hepatitis B virus (HBV) infection, hepatitis C virus (HCV) infection, alcohol consumption (AC), non-alcoholic fatty liver disease, and autoimmune liver disease [2]. Upon uncontrolled disease progression, these etiologies cumulated into LC—the end-stage of chronic liver disease [3], which is characterized by necrotizing inflammation and fibrotic process. Clinically, most LC patients died from decompensated LC and its complications for lacking applicable treatment strategies coupled with the poor compliance of patients [4]. Liver transplantation is an effective strategy for the treatment of LC [5], which is also limited by expensive expenditure and the risk of safety issues such as immune rejection and recurrent infections [6–8]. Therefore, it is of great urgency to develop effective treatment strategies for LC.

Mesenchymal stem cells (MSCs) are mainly derived from the early developmental mesoderm and widely exist in multiple body tissues such as the dermis (skin), synovial fluid, periosteum, blood, placenta, amniotic fluid, chorionic villi, muscle, dental pulp, breast milk, umbilical cords, and bone marrow [8–10]. MSCs are getting international consensus in the treatment of various tissue-damaging diseases due to their high self-renewal capacity and multi-directional differentiation potential [11, 12]. Compared with conventional treatments, the advantages of MSC transplantation therapy include low immunogenicity, immunomodulation, homing to the site of injury, tissue repair ability [13–15], and fewer ethical problems [16]. Recently, numerous animal studies have demonstrated the efficacy, safety, and feasibility of MSC in the treatment of LC [17–20]. More importantly, clinical trials have shown that infusion of MSCs can improve the indexes of liver function without obvious adverse effects [21–31]. Besides, studies have shown that MSCs also improved complications of LC such as ascites, hepatic encephalopathy, spontaneous bacterial peritonitis, and liver failure [32–34]. In conclusion, MSC transplantation is a potential strategy for LC treatment [6]. Indeed, some meta-analyses of MSC on liver disease had been conducted previously. Unfortunately, few studies have investigated the effects of different cell types in MSC treatment based on randomized controlled trials (RCTs). Furthermore, some factors should be considered, including cell dose, treatment frequency, and routes of transplantation, as well as the impact on LC complications and the incidence of Hepatocellular carcinoma (HCC). Herein, we screened and extracted data about MSCs for the treatment of LC in clinical trials and aimed to rigorously evaluate the efficacy and safety of MSC transplantation for LC through systematic evaluation and meta-analysis. Subgroup analyses depending on different treatment times, cell types, and cell doses were carried out. Collectively, these results might provide recommendations for the clinical application of MSC in LC treatment.

## Methods

The detailed agreement is registered in the PROSPERO. The registration numbers are CRD42023432691 (https://www.crd.york.ac.uk/PROSPERO/). This meta-analysis was carried out according to PRISMA guidelines (Additional file 3).

### Search strategies

The sources retrieved were mainly from published literature on the Web of Science, PubMed, EMBASE, and Cochrane Library, published in English. We systematically searched for eligible studies from the time the database was created until May 2023, using ‘‘mesenchymal stem cells’’ and ‘‘liver cirrhosis’’ as keywords. The detailed search method is shown in the Additional file 2. In addition, a further manual search of references was conducted to identify studies that met the inclusion criteria as a supplement. For example, we searched the references included in the study to prevent the omission of relevant literature.

### Study selection

Two authors (Long-xiang Yan and Wen-ming Lu) selected the literature that might meet the inclusion criteria by browsing the title, abstract, and keywords, respectively. We then obtained the full text after initial screening and subsequently evaluated the full text of potential studies to determine acceptability. If there are any differences, hold a meeting to resolve them. The criteria for inclusion in the study were to meet the PICOS (population, intervention, comparison, outcomes, and study design) principle, as shown below:

#### Inclusion criteria

*Population (P)*: Patients diagnosed with LC, regardless of country, region, or race.

*Intervention (I)*: Intervention is only MSC treatment.

*Comparison (C)*: Regular medication or placebo.

*Outcomes (O)*: Primary results: model for end-stage liver disease score (MELD); albumin (ALB); secondary outcomes: alanine aminotransferase (ALT); aspartate aminotransferase (AST); international normalized ratio (INR); total bilirubin (TBIL) hepatocellular carcinoma (HCC) survival rate.

*Study design (S)*: Only RCTs were included in this study.

#### Exclusion criteria

Conference abstracts, letters with duplicates, case reports, meta-analyses, reviews, non-English published literature, and incomplete or unavailable data were excluded. Moreover, studies not related to the topic of the article (such as studies using animal models and interventions that are not MSC transfusions) were excluded.

### Data extraction

Two reviewers (Xing-kun Tang and Wen-ming Lu) separately extracted the data from the included literature into a Microsoft Excel spreadsheet and then summarized the data into a table. Any differences were resolved through serious discussions. The following information was extracted from the included literature: study characteristics (publication year, first author, research area), study patient characteristics (number of enrolled patients, stage of LC, cause of LC, follow-up time), details of intervention (type of cells, cell dosage, administration route), main outcomes measures and different follow-up time point.

### Assessment of the risk of bias in the included studies

Two reviewers (Long-xiang Yan and Jia-yang Qu) independently evaluated the risk of bias for each included study according to the Cochrane evaluation tools [35]. Disagreements were resolved by two reviewers discussing or consulting with a third author (Xue-song Wang), if necessary. The following items were evaluated: generation of randomized outcomes, allocation concealment, blinding of participants, investigators and outcome assessment, completeness of outcome information, selective reporting, and other sources of bias. Each item was classified as low risk, high risk, or unclear risk.

### Statistical analyses

Review Manager 5.4 software was used to perform data analysis of the overall and subgroup treatment effects of MSC interventions in LC. The data needed for meta-analysis were extracted directly from the original literature or calculated indirectly based on the original data through transformation tools. Then, the pre-extracted mean, standard deviation, and sample size were entered into the analysis software. In our meta-analysis, for studies using the same measures, we used mean differences (MD) to report effect sizes. However, for studies using different time points measuring the same outcome, the standardized mean difference (SMD) was used to report continuous outcomes. In addition, the *I*^2^ statistic was used to analyze heterogeneity between studies. If the *p*-value of the heterogeneity test was less than 0.05, we used the random-effects model; Otherwise, a fixed-effects model was used. For results with high heterogeneity between the two groups, sensitivity analysis or subgroup analysis was used to assess the outcome; if the sources of heterogeneity could not be analyzed, a descriptive analysis was carried out. Statistical significance was demonstrated by *p* < 0.05.

## Results

### Results of the search

**Fig. 1:**
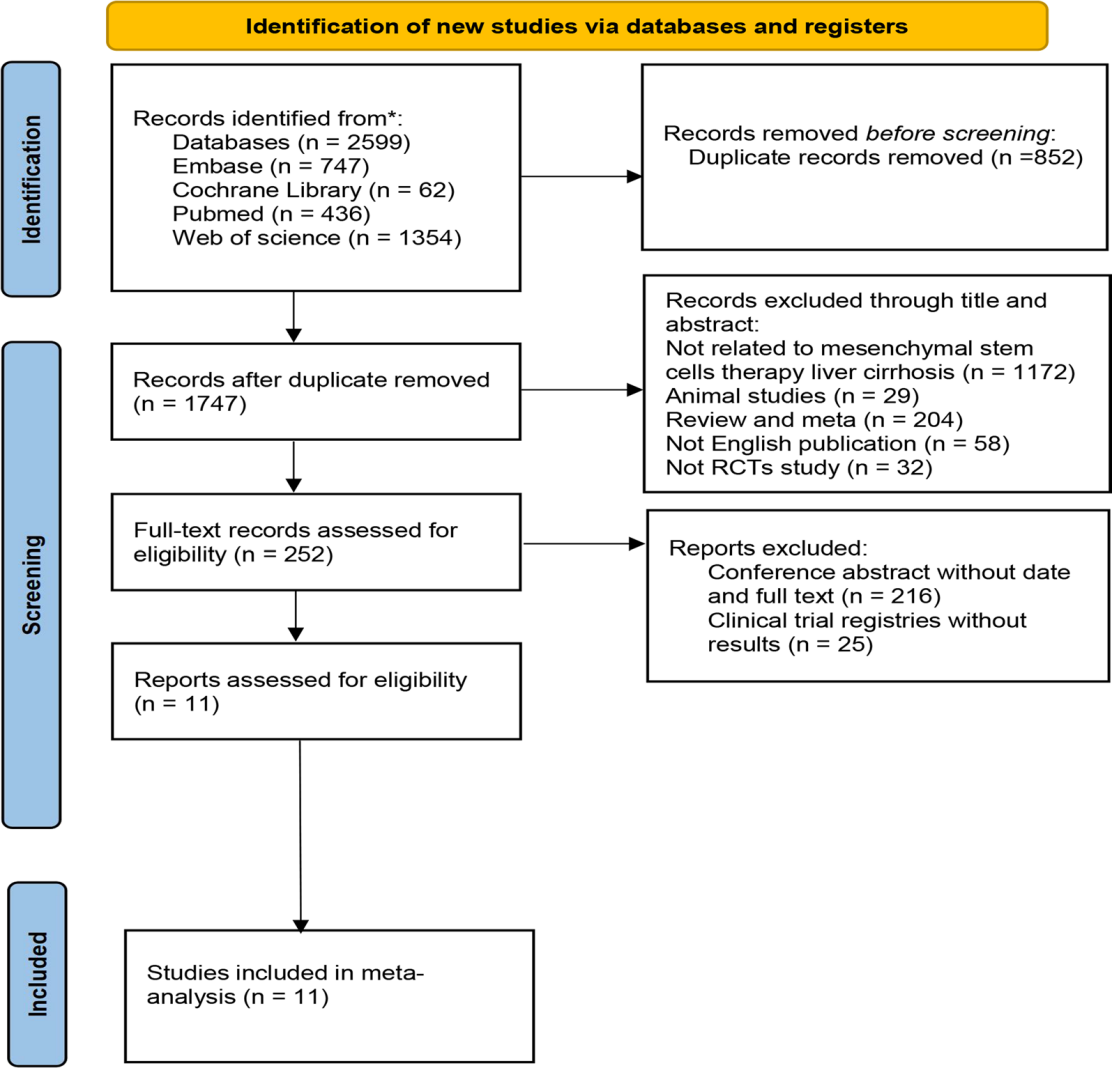
Literature selection and inclusion process

After a systematic search of 4 databases, we retrieved a total of 2599 articles, including 436 in PubMed, 747 in Embase, 1354 in Web of Science, and 62 in Cochrane Library. After screening out duplicates, 1747 articles were left. After reading the title abstracts, 1172 articles were excluded due to no relevant topics, 29 articles were animal experimental models, 204 articles were previous reviews and meta, 32 studies were not RCTs, 58 articles were published without in English, 252 potentially eligible articles were included. However, when the full text was reviewed, 216 articles were conference abstracts without full text and relevant data, and 25 studies were clinical trial registries without results. As a result, 11 articles were included in our meta-analysis. The detailed screening and inclusion process is shown in Fig. 1.

### Characteristics of the studies

Among the eleven included studies, two studies recruited patients from Iran and Korea respectively. Six of the remaining nine studies came from China and three from Egypt. All studies were randomized controlled trials. Of these, four studies infused cells from umbilical cord MSCs (UC-MSCs), while the rest of the studies were bone marrow MSCs (BM-MSCs). All the studies’ sample sizes ranged from 10 to 111 and were published from 2011 to 2023. The main transfused routes were intravenous infusion and hepatic artery injection, in which the total dose ranged from 4 × 10^5^ to 3.9 × 10^7^ cells/kg. The etiology of LC is mainly HBV/HCV. Table 1 presents detailed characteristics of the included studies.

**Table 1:** Summary of clinical studies of mesenchymal stem cell therapy for liver cirrhosis

Included studies	Study period	Country	Stage of LC	Design	Age(year)	Patients(con/exp)	Cell type	Cell dose	Total dose/kg body weight (average 50 kg/person)	Administration route	Follow up	Main outcome measures	Cause of LC
Mohamadnejad et al. [23]	2007.7–2010.8	Iran	ND	RCT	18–65	11, 14	BM-MSCs	195 × 10^7^cells	3.9 × 10^7^cells/kg	Cubital vein of the arm	3,12 months	MELD score, Child–Pugh, Score, ALB, INR, AST, ALT,	HBV/HCV
Fang et al. [22]	2013.5–2017.3	China	ND	RCT	18–75	53, 50	HUCB-MSCs	(4.0–4.5) × 10^8^ cells two times	(1.6–1.8) × 10^7^cells/kg	Intravenous	2,4,8,12,24,36, 48 weeks	ALT, AST, ALB, TBIL, PT, MELD core, Child–Pugh score	HBV
Suk et al. [26]	2013.1–2015.11	Korea	Child–Pugh B-C	RCT	20–70	18, 18, 19	BM-MSCs	5 × 10^7^ cells /ml 10 ml two Times	1 × 10^7^cells/kg	Hepatic artery	3, 6 months	AST, ALT, TBIL, ALP, GGT, INR, MELD core, Child–Pugh score, ALB	AC
Salama et al. [25]	2010.6–2011.10	Egypt	ND	RCT	20–60	20, 20	BM-MSCs	1 × 10^6^cells/kg body weight single time	1 × 10^6^cells/kg	Intravenous	2 week,1,3,6 month	Bilirubin, ALB, PC, INR, AST, ALT,	HCV
El-Ansary et al. [21]	ND	Egypt	Child–Pugh C	RCT	30–60	10, 15	BM-MSCs	1 × 10^6^cells/kg body weight	1 × 10^6^cells/kg	Intravenous	3,6 months	ALB, TBIL, MELD score	HCV
Peng et al. [24]	2005.5–2009.6	China	ND	RCT	15–75	105, 53	BM-MSCs	10 ml	UK	Hepatic artery	1,2,3,4,12, 24,36,48 weeks	ALT, ALB, TBIL, PT, MELD score	HBV
Bai et al. [28]	2009.3–2011.3	China	Child–Pugh B-C	RCT	ND	15, 32	BM-MNCs	1.0–11.2 × 10^7^cells/ml. 10 ml/h	UK	Hepatic artery	1 week,1,3,6,12,24 months	TBIL, ALB	HBV/AC
Amer et al. [27]	2008.10–2009.6	Egypt	Child–Pugh C	RCT	45–60	20, 20	BM-MSCs	2 × 10^7^ cells, single time	4 × 10^5^ cells/kg	Hepatic artery	2 weeks,1,2,4, 6 months	Child–Pugh score, MELD score	HCV
Li et al. [29]	2010.11–2013.2	China	ND	RCT	18–75	72, 36	HUCB-MSCs	ND	UK	ND	3-year, 5-year	3-year and 5-year SR	ND
Shi et al. [30]	2010.10–2017.10	China	ND	RCT	18–65	111, 108	HUC-MSCs	5 × 10^5^cells/kg body weight	5 × 10^5^cells/kg	Intravenous	2, 4, 8, 12,24,48 week, 24, 48, 60, 75 months	OSR, HCC-free SR	HBV
Zhang et al. [31]	ND	China	ND	RCT	25–64	15, 30	HUC-MSCs	5 × 10^5^cells/kg body weight	5 × 10^5^cells/kg	Intravenous	0, 1, 2, 4, 8, 12, 24,36,48 weeks	TBIL, CHE, PTA, INR	HBV

Con: control group; Exp: experimental group; LC: liver cirrhosis; ND: non-determined; UK: unknown; RCT: randomized controlled trial; BM-MSCs: bone marrow mesenchymal stem cells; BM-MNCs: bone marrow mononuclear cells; HUC-MSCs: human umbilical cord mesenchymal stem cells; HUCB-MSCs: human umbilical cord blood mesenchymal stem cell; MELD: model for end-stage liver disease; ALB: albumin; ALT: alanine aminotransferase; AST: aspartate aminotransferase; INR: international normalized ratio; TBIL: total bilirubin: PT: prothrombin time; ALP: alkaline phosphatase; GGT: gamma-glutamyl transferase; PC: prothrombin concentration; HBV: hepatitis B virus; HCV: hepatitis C virus: AC: alcohol consumption; OSR: overall survival rate; SR: survival rate; CHE: cholinesterase; PTA: prothrombin activity

### Risk assessment of bias

**Fig. 2:**
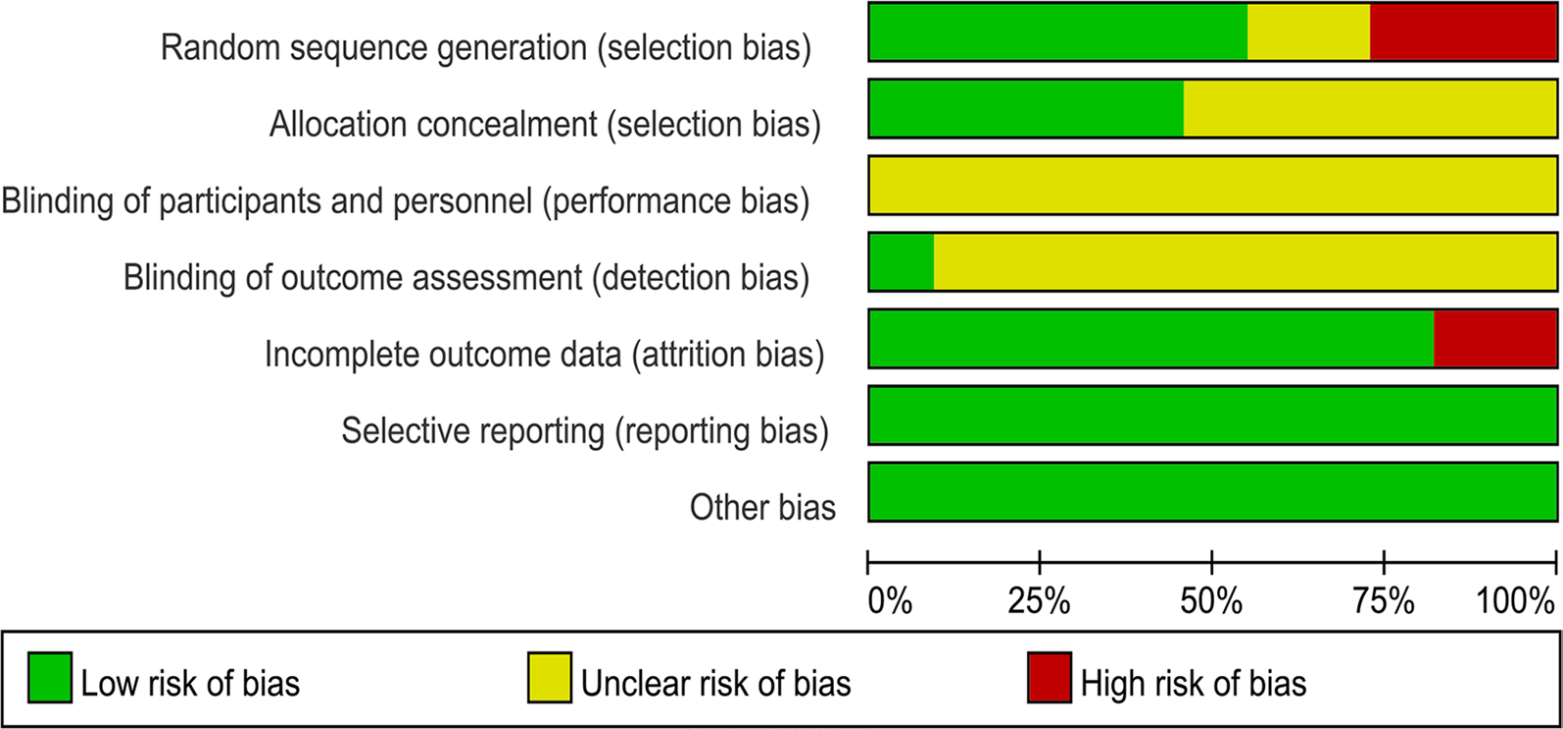
Risk of bias graph: review authors' judgments about each risk of bias item presented as percentages across all included studies

**Fig. 3:**
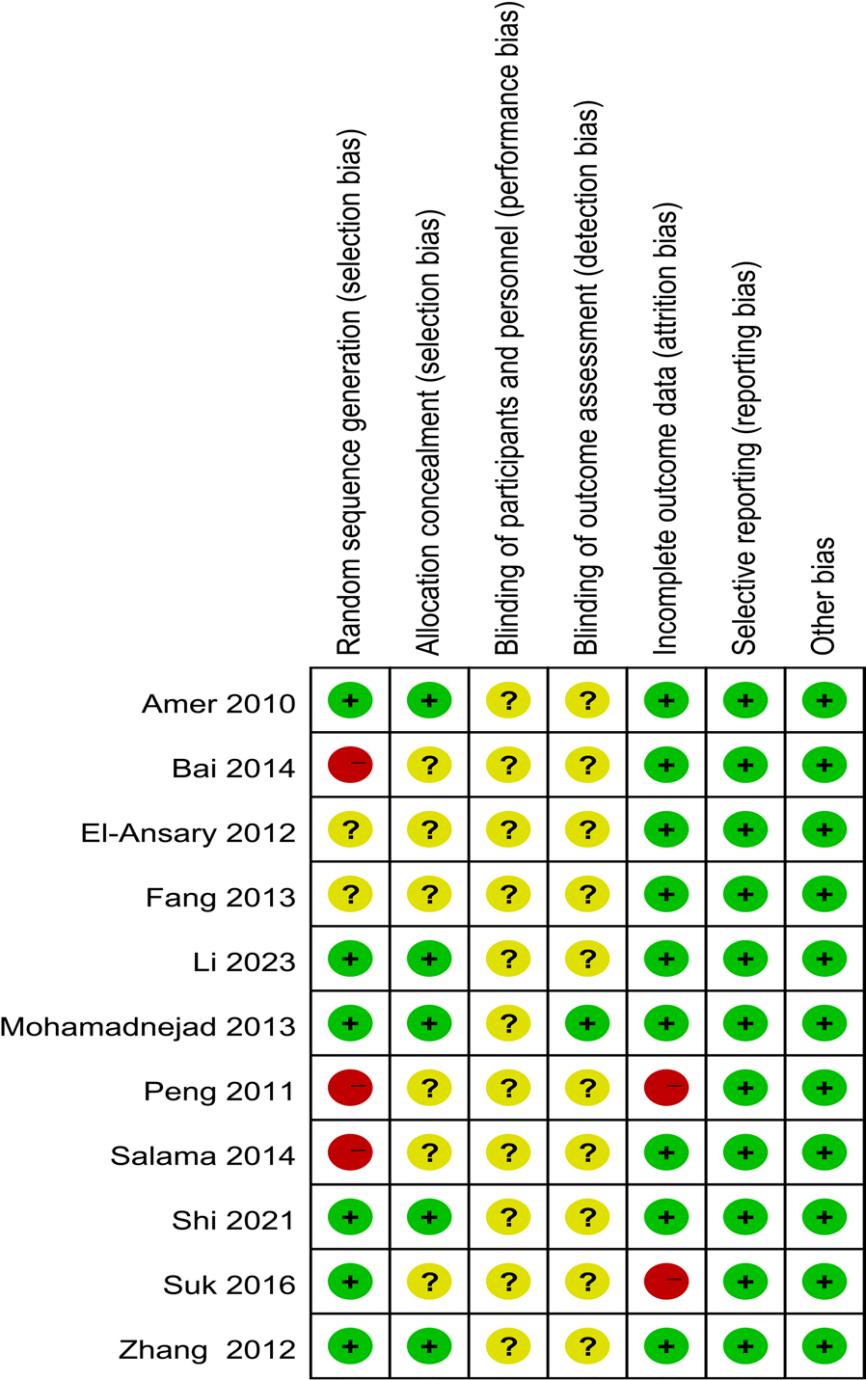
Risk of bias summary: review authors' judgments about each risk of bias item for each included study

The assessment results of bias risk and methodological suitability of the included studies were presented in Figs. 2 and 3. We found that random outcome generation was high risk in three studies [24, 25, 28], five studies were low risk of bias [23, 26, 27, 29–31] and random outcome generation was not mentioned in two studies [21, 22] (unclear risk of bias). Allocation concealment was mentioned in five studies [23, 27, 29–31] (low risk of bias) and was not mentioned in six studies (unclear risk of bias). For the blinding of the outcome assessment, one retrieved study was low risk [23] and ten were not reported (risk of bias unclear). Most studies had complete information results (low risk of bias), and only two studies reported patients who dropped out but were not included in the analysis (high risk of bias). All studies were free from selective reporting bias and other biases. Funnel plot analysis was not performed due to insufficient included studies.

### Meta-analysis

Eleven eligible articles were included for meta-analysis using a random-effects model and descriptive analysis, with MELD score and ALB as primary and ALT, AST, TBIL, and INR as secondary indicators to evaluate the effectiveness of MSC for LC, and adverse events (AE) as a safety indicator.

#### Primary indicators

##### MELD score

**Fig. 4:**
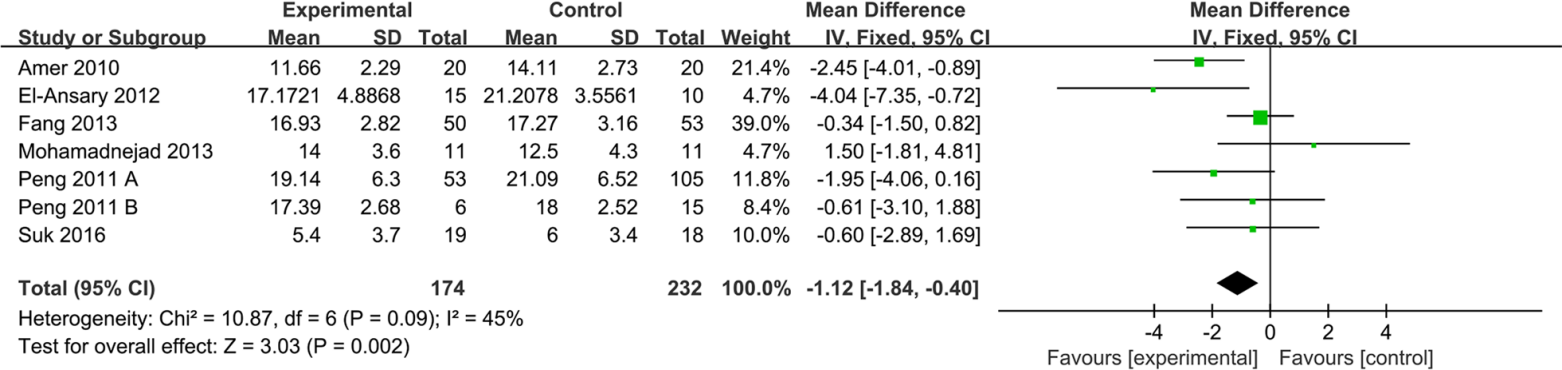
Forest plot of primary indicator: pooled results of MELD score

MELD score was reported in 6 studies of 174 patients in the MSC group and 232 patients in the control group. We found that the MELD score scale was consistent for each study, so WMD was selected as the effect indicator (Fig. 4). The MELD score of the six studies showed a statistically significant effect size (WMD =  − 1.12; 95% CI = [− 1.84, − 0.40]; *p* = 0.002; *I*^2^ = 45%; heterogeneity test *I*^2^ = 45%; *p* = 0.09) (Fig. 4). However, to investigate the effect of various factors on the efficacy of MSCs therapy on MELD score, the subgroup analysis of MELD score was performed.

##### ALB levels

**Fig. 5:**
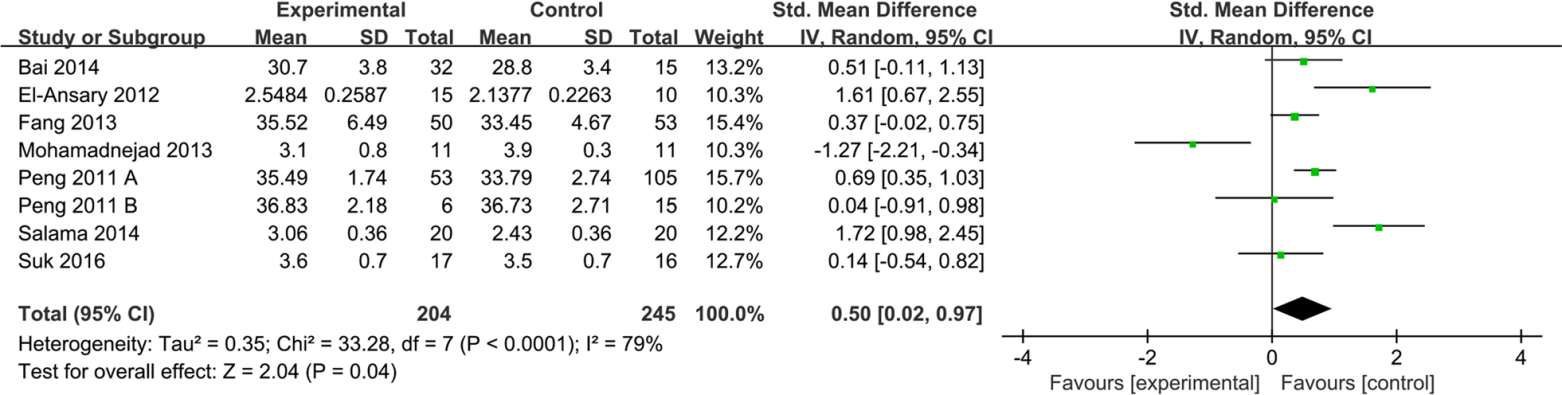
Forest plot of primary indicator: pooled results of ALB levels before sensitivity analysis

**Fig. 6:**
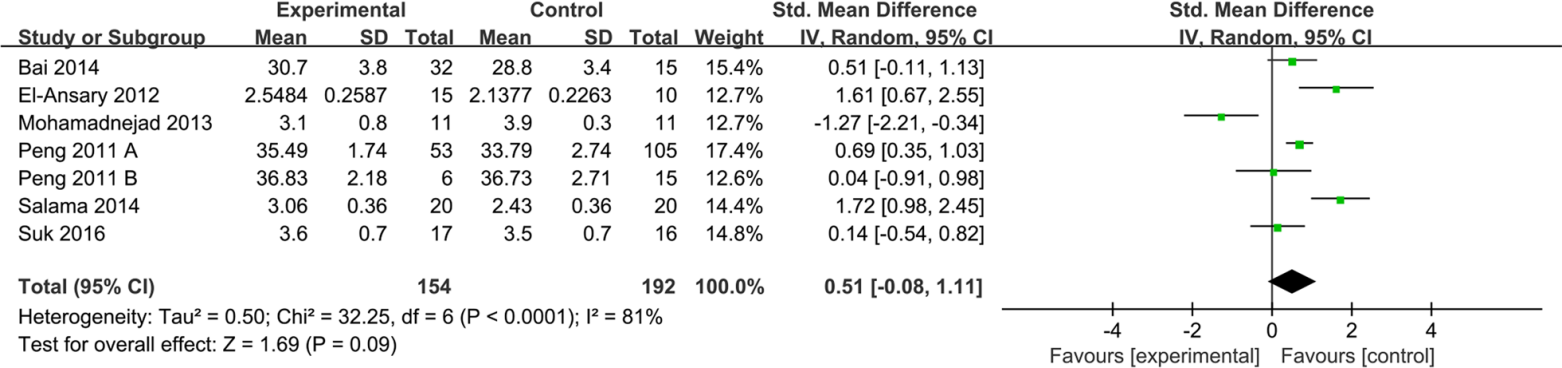
Forest plot of primary indicator: pooled results of ALB levels after eliminating heterogeneous

ALB levels were reported in 7 studies of 204 patients in the MSC group and 245 patients in the control group. Due to the large difference in mean values between studies. Therefore, the random effects model was adopted as the effect indicator (Fig. 5). The forest map results showed that statistically significant differences between the two groups were observed (SMD: 0.50; 95% CI [0.02, 0.97]; *p* = 0.04; heterogeneity test *p* < 0.0001; *I*^2^ = 79%) (Fig. 5). To investigate the effect of various factors on the efficacy of MSCs, the subgroup analysis of ALB levels was performed (Fig. 6).

#### Subgroup of model for end-stage liver disease

##### Time subgroup of model for end-stage liver disease

**Fig. 7:**
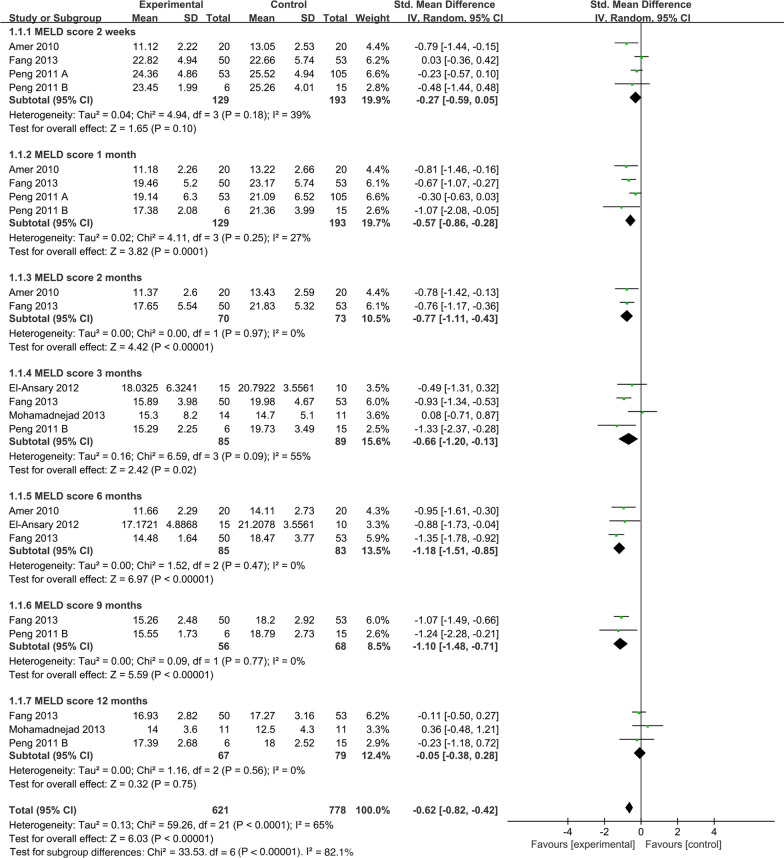
Time subgroup of MELD score

To explore the effect of MSC infusion on MELD score at different time points after infusion, we conducted a time subgroup analysis for MELD score (Fig. 7). Pooled analysis showed that the MSC group significantly decreased MELD score (SMD =  − 0.62; 95% CI [− 0.82, − 0.42]; *p* < 0.00001; heterogeneity test *p* < 0.0001; *I*^2^ = 65%), compared with the control group. Subgroup analysis with random-effects model showed that the MSC group significantly decreased MELD score in 1 month (SMD: − 0.57; 95% CI [− 0.86, − 0.28]; *p* = 0.0001), 2 months (SMD: − 0.77; 95% CI [− 1.11, − 0.43]; *p* < 0.00001), 3 months (SMD: − 0.66; 95% CI [− 1.20, − 0.13]; *p* = 0.02), heterogeneity test *p* = 0.09; *I*^2^ = 55%), 6 months (SMD: − 1.18; 95% CI [− 1.51, − 0.85]; *p* < 0.00001), and 9 months (SMD: − 1.10; 95% CI [− 1.48, − 0.71]; *p* < 0.00001). However, comparisons between the MSC group and the control group showed no difference in 2 weeks (SMD: − 0.27; 95% CI [− 0.59, − 0.05]; *p* = 0.10) and in 12 months (SMD: − 0.05; 95% CI [− 0.38, 0.28]; *p* = 0.75; heterogeneity test *p* = 0.56; *I*^2^ = 0%) (Fig. 7).

##### Administration route subgroup of model for end-stage liver disease

**Fig. 8:**
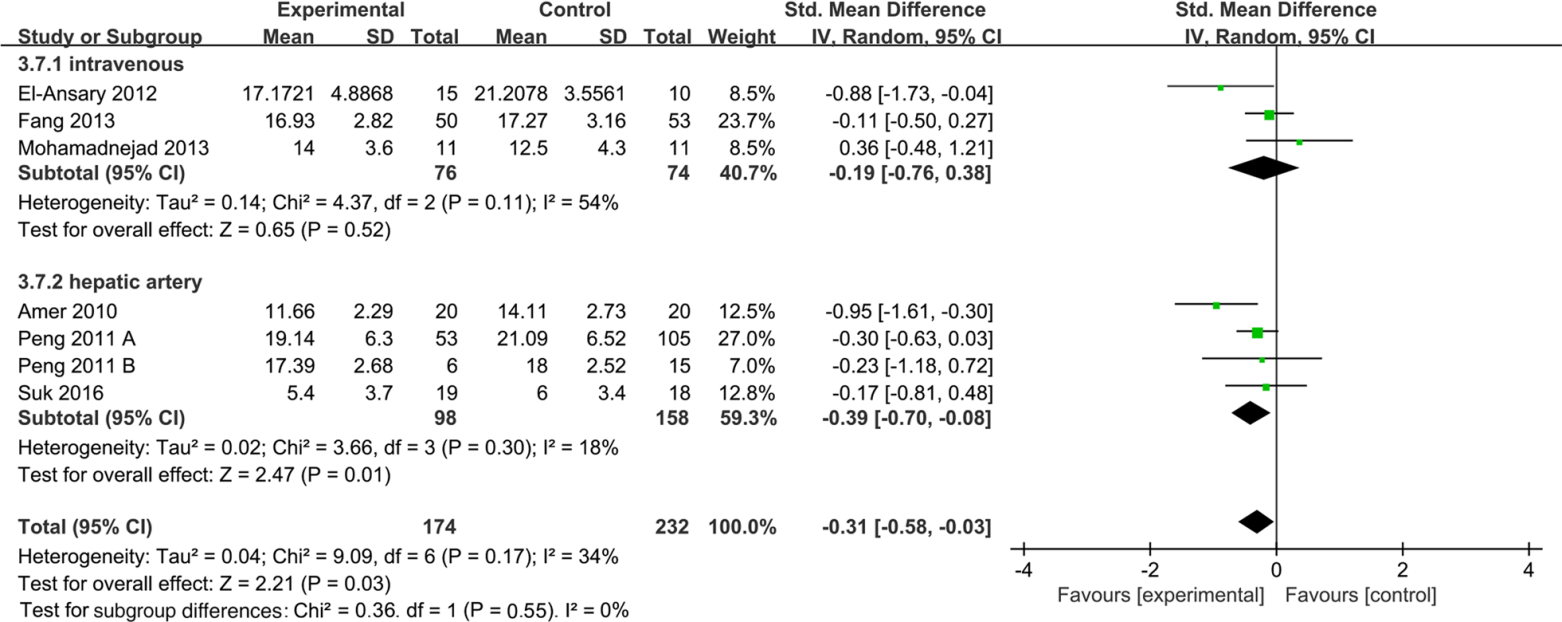
Forest plot of subgroup in MELD score: Forest plot demonstrating the effect of MSCs compared with controls in subgroup of administration route

In the included literature, the main methods of MSC transplantation included intravenous and hepatic arterial injections. Pooled analysis showed that the MSC group significantly decreased MELD score (SMD =  − 0.31; 95% CI [− 0.58, − 0.03]; *p* = 0.03; heterogeneity test *p* = 0.17; *I*^2^ = 34%), compared with the control group (Fig. 8). Subgroup analysis with random effects model presented that the MSC group significantly decreased MELD score in the hepatic artery route (SMD =  − 0.39; 95% CI [− 0.70, − 0.08]; *p* = 0.01; heterogeneity test *p* = 0.30; *I*^2^ = 18%). However, no statistically significant differences were observed in the intravenous subgroup (SMD =  − 0.19; 95% CI [− 0.76, 0.38]; *p* = 0.52; heterogeneity test *p* = 0.11; *I*^2^ = 54%) (Fig. 8).

#### Subgroup of albumin levels

##### Time subgroup of albumin levels

**Fig. 9:**
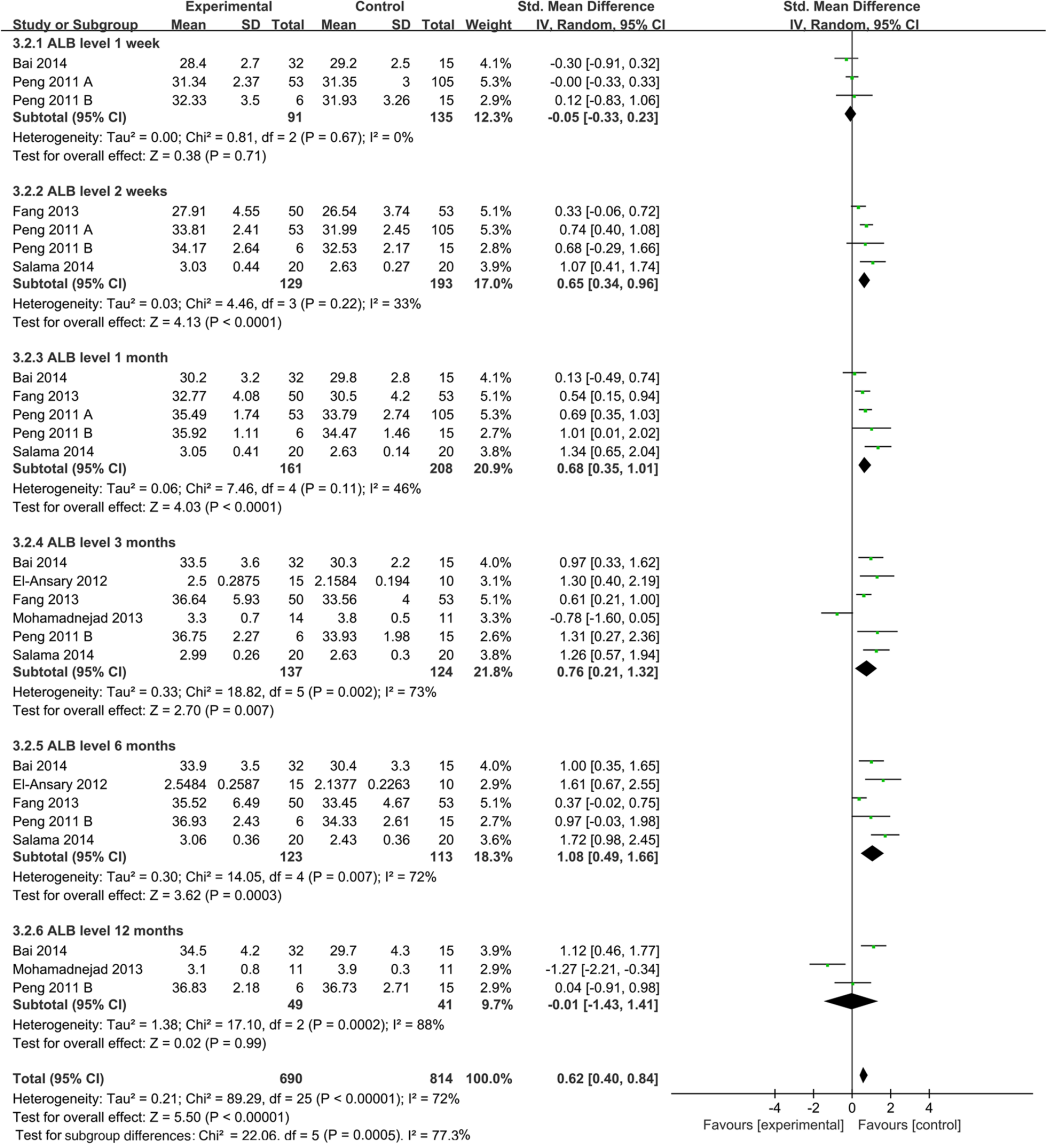
Time subgroup of ALB levels

To explore the effect of MSC infusion on ALB levels at different time points after infusion, the time subgroup analysis for ALB levels was performed. Pooled analysis showed that ALB levels of the MSC group were significantly improved (SMD = 0.62; 95% CI [0.40, 0.84]; *p* < 0.00001; heterogeneity test *p* < 0.00001; *I*^2^ = 72%) compared with the control group (Fig. 9). Subgroup analysis with random-effects model showed that the MSC group significantly increased ALB levels in 2 weeks (SMD = 0.65; 95% CI [0.34, 0.96]; *p* < 0.0001), 1 month (SMD: 0.68; 95% CI [0.35, 1.01]; *p* < 0.0001), 3 months (SMD: 0.76; 95% CI [0.21, 1.32]; *p* = 0.007; heterogeneity test *p* = 0.002; *I*^2^ = 73%), and 6 months (SMD = 1.08; 95% CI [0.49, 1.66]; *p* = 0.0003; heterogeneity test *p* = 0.007; *I*^2^ = 72%). Whereas, comparisons between the two groups showed no statistical differences in 1 week (SMD =  − 0.05; 95% CI [− 0.33, 0.23]; *p* = 0.71), and 12 months (SMD =  − 0.01; 95% CI [− 1.43, 1.41]; *p* = 0.99; heterogeneity test* p* = 0.0002; *I*^2^ = 88%) (Fig. 9).

##### Administration route subgroup of albumin levels

**Fig. 10:**
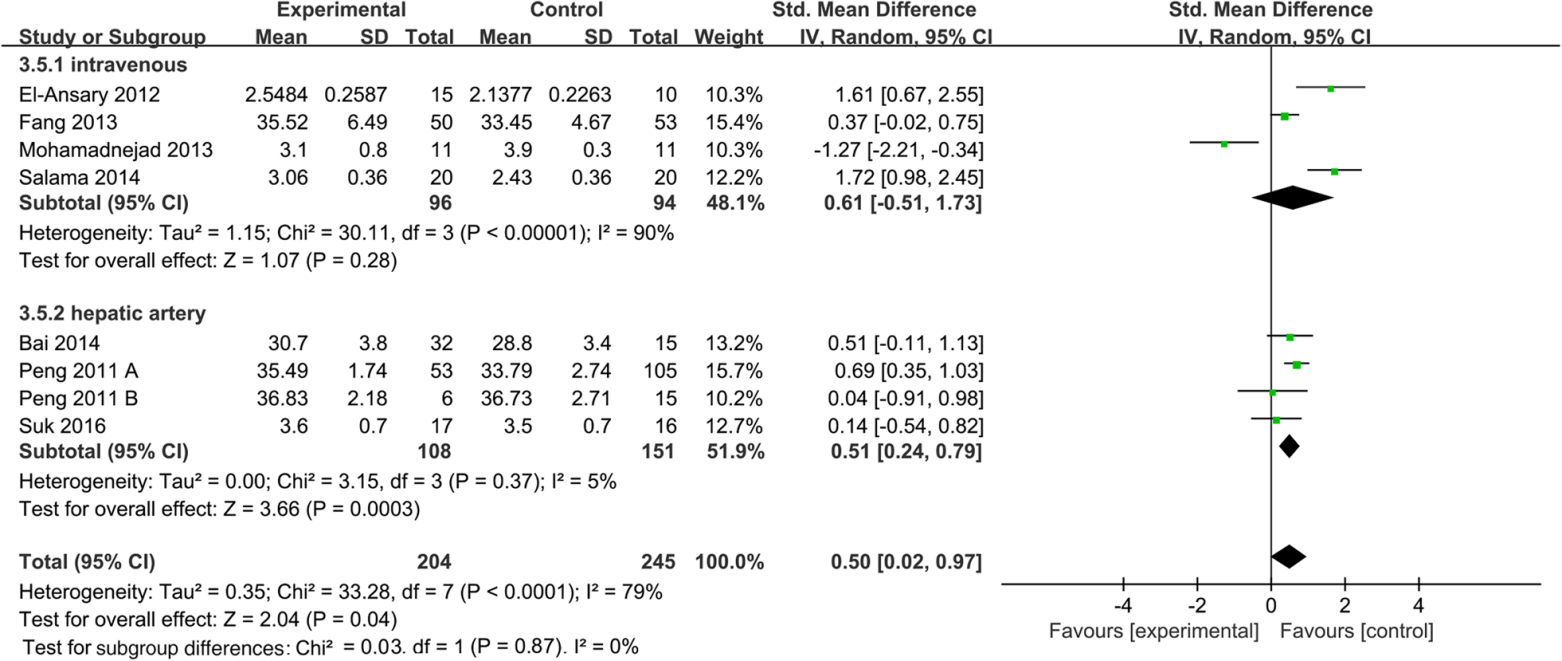
Administration route subgroup of ALB levels

In the included literature, the main methods of MSC transplantation included intravenous and hepatic arterial injections. Pooled analysis showed that the MSC group significantly increased ALB levels (SMD = 0.50; 95% CI [0.02, 0.97]; *p* = 0.04; heterogeneity test *p* < 0.0001; *I*^2^ = 79%) compared with the control group (Fig. 10). Subgroup analysis with random effects model presented that the MSC group significantly increased ALB levels in the hepatic artery route (SMD = 0.51; 95% CI [0.24, 0.79]; *p* = 0.0003; heterogeneity test *p* = 0.37; *I*^2^ = 5%). However, no statistically significant differences were observed in the intravenous subgroup (SMD = 0.61; 95% CI [− 0.51, 1.73]; *p* = 0.28; heterogeneity test *p* < 0.00001; *I*^2^ = 90%) (Fig. 10).

##### Cause of disease subgroup of albumin levels

**Fig. 11:**
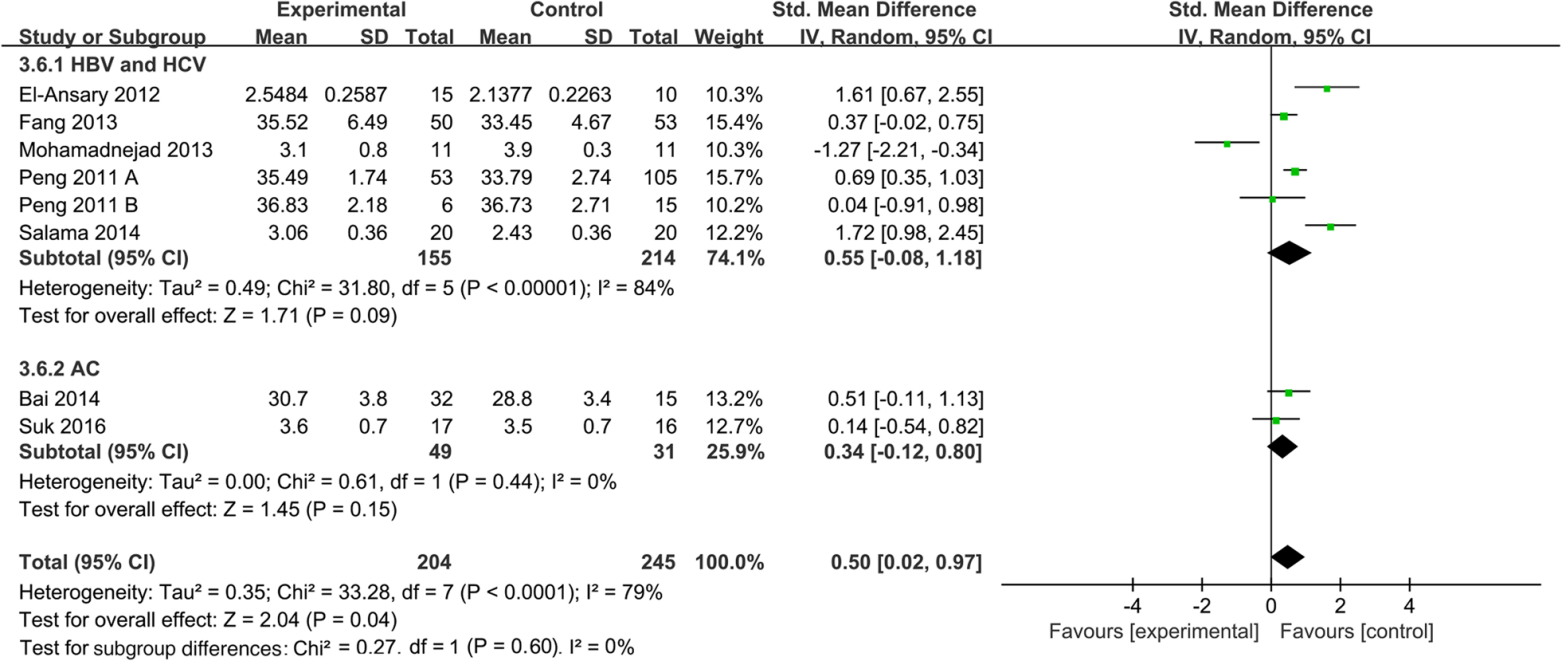
Causes of disease subgroup of ALB levels

To explore the effect of different etiologies on the efficacy of mesenchymal stem cells. We performed the etiologies subgroup analysis for ALB levels (Fig. 11). Data showed that the ALB levels of the treatment group exerted no statistical differences from the control group both in the HBV-HCV group (SMD: 0.55; 95% CI [− 0.08, 1.18]; *p* = 0.09; heterogeneity test *p* < 0.00001; *I*^2^ = 84%). and in the AC group (SMD: 0.34; 95% CI [− 0.12, 0.80]; *p* = 0.15; heterogeneity test *p* = 0.44; *I*^2^ = 0%). Significant heterogeneity between the two etiologies was found (heterogeneity test *p* < 0.0001; *I*^2^ = 79%) (Fig. 11).

##### Dose subgroup of albumin levels

**Fig. 12:**
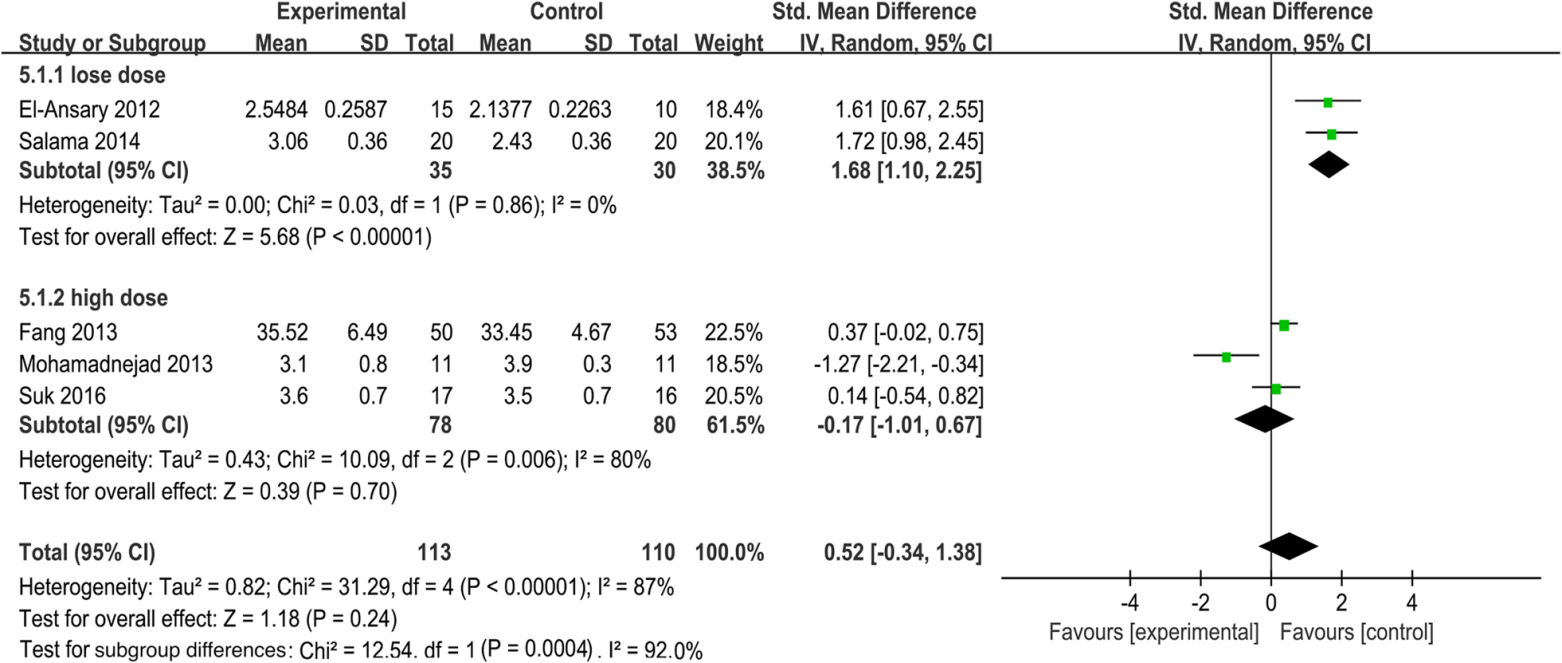
Dose subgroup of ALB levels

To explore the effect of different cell doses on the efficacy of mesenchymal stem cells. We performed the dose subgroup analysis for ALB levels (Fig. 12). Studies were divided into two grades based on the total number of cells injected: low-dose (1 × 10^6^ cells/kg), and high-dose (1.0 × 10^7^–3.9 × 10^7^ cells/kg). Pooled analysis showed that the MSC group had no significant increase in ALB levels (SMD = 0.52; 95% CI [− 0.34, 1.38]; *p* = 0.24) compared with the control group. Subgroup analysis with a random-effects model showed significantly increased ALB levels in the low-dose (SMD = 1.68; 95% CI [1.10, 2.25]; *p* < 0.00001) subgroup but not in the high-dose (SMD =  − 0.17; 95% CI [− 1.01, 0.67]; *p* = 0.70; heterogeneity test *p* = 0.006; *I*^2^ = 80%) subgroup (Fig. 12).

#### Secondary indicators

##### ALT levels

In this systematic review, ALT levels were reported in 5 studies of 157 patients in the MSC group and 220 patients in the control group. The meta-analysis showed a non-significant pooled effect size between the two groups (WMD: 0.21; 95% CI [− 0.44, 0.86]; *p* = 0.52; heterogeneity test *p* = 0.35; *I*^2^ = 10%) (Additional file 1: Fig. S1A). We carried out a subgroup analysis to make the conclusions more accurate. For the time subgroup of ALT levels, the pooled analysis presented that there were no differences in ALT levels (SMD =  − 0.09, 95% CI [− 0.30, 0.11], *p* = 0.38, heterogeneity test *p* = 0.03; *I*^2^ = 47%). Similarly, subgroup analysis with random-effects model showed that no significant differences were observed in 2 weeks (SMD =  − 0.16, 95% CI [− 0.57, 0.25], *p* = 0.44), 1 month (SMD: − 0.12; 95% CI [− 0.54, 0.30]; *p* = 0.57; heterogeneity test* p* = 0.04; *I*^2^ = 63%), 3 months (SMD: − 0.12; 95% CI [− 0.55, 0.31]; *p* = 0.59), 12 months (SMD: 0.35; 95% CI [− 0.39, 1.09]; *p* = 0.35) (Additional file 1: Fig. S2).

##### AST levels

In this meta-analysis, AST levels were reported in 4 studies of 98 patients in the MSC group and 100 patients in the control group. The forest map results show that no statistically significant differences were found between the two groups (WMD: 0.46; 95% CI [− 0.02, 0.95]; *p* = 0.06; heterogeneity test *p* = 0.11; *I*^2^ = 49%) (Additional file 1: Fig. S1B). We carried out a subgroup analysis to make the conclusions more accurate. For the time subgroup of AST levels, a pooled analysis showed that there were no differences between the two groups (SMD =  − 0.06; 95% CI [− 0.30, 0.19]; *p* = 0.65; heterogeneity test *p* = 0.03; *I*^2^ = 52%). Subgroup analysis with a random-effects model showed that no significant differences were observed between the two groups in 2 weeks (SMD = 0.09; 95% CI [− 0.57, 0.75]; *p* = 0.80; heterogeneity test *p* = 0.07; *I*^2^ = 69%), 1 month (SMD =  − 0.20; 95% CI [− 0.61, 0.20]; *p* = 0.32),3 months (SMD =  − 0.08; 95% CI [− 0.60, 0.43]; *p* = 0.75; heterogeneity test *p* = 0.10; *I*^2^ = 56%), and 6 months (SMD = 0.09; 95% CI [− 0.87, 1.06]; *p* = 0.85; heterogeneity test *p* = 0.010; *I*^2^ = 85%) (Additional file 1: Fig. S3).

##### TBIL levels

TBIL levels were reported in 6 studies of 193 patients in the MSC group and 234 patients in the control group. The data showed that no statistically significant differences were found between the two groups (WMD: − 0.19; 95% CI [− 0.50, 0.13]; *p* = 0.24; heterogeneity test *p* = 0.06; *I*^2^ = 51%) (Additional file 1: Fig. S1C). To make the conclusions more accurate, we carried out a subgroup analysis. For the time subgroup of TBIL levels, the pooled analysis showed that the MSC group significantly decreased TBIL levels (SMD =  − 0.36; 95% CI [− 0.49, − 0.22]; *p* < 0.00001; heterogeneity test *p* = 0.11; *I*^2^ = 27%), compared with the control group. Subgroup analysis with random-effects model showed that the MSC group significantly improved TBIL levels in 1 month (SMD =  − 0.33; 95% CI [− 0.55, − 0.12]; *p* = 0.002), 3 months (SMD =  − 0.64; 95% CI [− 0.91, − 0.37]; *p* < 0.00001), and 6 months (SMD =  − 0.45; 95% CI [− 0.88, − 0.01]; *p* = 0.04; heterogeneity test *p* = 0.07; *I*^2^ = 55%). However, the comparison between the two groups showed that no significant changes were found in 1 week (SMD =  − 0.02; 95% CI [− 0.30, − 0.26]; *p* = 0.89), 2 weeks (SMD =  − 0.22; 95% CI [− 0.45, 0.00]; *p* = 0.05), and 12 months (SMD =  − 0.22; 95% CI [− 1.37, 0.92]; *p* = 0.70; heterogeneity test *p* = 0.05; *I*^2^ = 75%) (Additional file 1: Fig. S4).

##### INR levels

INR levels were reported in 3 studies of 48 patients in the MSC group and 47 patients in the control group. The data showed that no statistically significant differences were found between the two groups (WMD: − 0.13; 95% CI [− 0.73, 0.48]; *p* = 0.68; heterogeneity test *p* = 0.12; *I*^2^ = 53%) (Additional file 1: Fig. S1D). The time subgroup of INR could not be carried out due to the inconsistent follow-up time points of the 3 studies.

#### Sensitivity analysis

To test the stability and reliability of the meta-analysis results, the sensitivity analysis of the main outcome indicators was carried out. Forest plots of ALB levels showed heterogeneity test *p* < 0.0001; *I*^2^ = 79% (Fig. 5). The study performed by Fang et al. in which stem cells were extracted from the umbilical cord, might be the cause of heterogeneity [22]. We found that the result of ALB levels became meaningless and higher heterogeneity when the study was removed (SMD = 0.51; 95% CI [− 0.08, 1.11]; *p* = 0.09; heterogeneity test *p* < 0.0001; *I*^2^ = 81%) (Fig. 6), which related to inconsistency in follow-up period between studies, causing publication bias into the conclusions.

#### Descriptive analysis

##### Complications of LC

Among the included studies, 4 studies suggested significant improvements in the ascites of participants compared to the baseline [21, 25, 27, 31]. El-Ansary et al. and Zhang et al. consistently found that MSC infusion significantly reduced ascites volume in decompensated LC patients relative to the control group (*p* < 0.05). Moreover, MSC also improves complications of LC, such as hepatic encephalopathy, spontaneous bacterial peritonitis, and liver failure [21, 24, 25, 27]. Salama et al. showed that the incidence of hepatic encephalopathy was decreased in the MSC group in comparison with the control group.

##### Hepatocellular carcinoma

Shi et al. reported that UC-MSC treatment improved survival in patients with decompensated LC and did not increase the frequency of HCC events [30]. Similarly, Li et al. announced that HUCB-MSC extended the long-term survival without increasing the risk of HCC in patients with decompensated LC [29]. Importantly, Peng et al. documented that MSC transplantation exerted protective effects on HCC incidence and mortality in compensated LC patients.

##### Fibrosis biomarkers

Salama et al. reported that MSC infusion reduces serum levels of procollagen III C terminal peptide (PIIICP) and procollagen III N-terminal peptide (PIIINP) in LC patients compared with the control group (*p* < 0.05) [25]. Zhang et al. also found that the concentrations of fibrotic markers, such as serum laminin, hyaluronic acid, PIIINP, and type IV collagen, were significantly decreased after 24 and 48 weeks in the UC-MSC treatment group [31].

#### Adverse events assessment

Safety should be a key concern after the treatment of MSCs. We assessed the adverse effects that occurred after treatment. Serious adverse reactions were rare, and fever was the most common condition, which subsided by regular antipyretics (Table 2).

**Table 2:** Information of adverse effects after the MSCs infusion

Included studies	Adverse effects (events)
Mohamadnejad et al. [23]	ND
Fang et al. [22]	No obvious adverse reactions
Suk et al. [26]	No obvious adverse reactions
	1 case of fever
Salama et al. [25]	ND
El-Ansary et al. [21]	ND
Peng et al. [24]	ND
Bai et al. [28]	No obvious adverse reactions
Amer et al. [27]	10 cases of fever
	3 cases of transient shivering
Zhang et al. [31]	4 cases of a self-limiting fever
Shi et al. [30]	7 cases of a self-limiting fever
Li et al. [29]	ND

MSCs, mesenchymal stem cells; ND, non-determined

## Discussion

LC is a progressive liver disease, and there are no clinical drugs for the treatment of LC. Liver transplantation is an effective treatment approach for LC. However, the broad clinical application of liver transplantation in LC is impeded by limitations such as demanding surgical indications and expensive costs [36]. Recently, mountains of evidence have presented the potential of MSC in clinical application. For instance, the animal model experiment showed that a 7-day consecutive tail vein injection of UC-MSCs significantly improved liver function in carbon tetrachloride-treated mice [37]. Currently, eleven RCTs were enrolled to conduct a meta-analysis and systematic evaluation of the efficacy and safety of MSC in the treatment of LC. The results showed that MSC infusion significantly improved liver function in LC patients, as was indicated by the reduced MELD score and increased ALB levels. Remarkably, no significant adverse effects were reported in the included studies, which suggested the safety of MSC therapy for LC. In addition, further analysis based on the following research might provide more valuable references for clinical trial design in the future.

Our primary concern is the duration of MSC therapy. According to the enrolled research, patients of decompensated LC are frequently accompanied by comorbidities including appetite loss, mental depression, and jaundice linked to liver failure, which lowers scores of patient quality of life and negatively affects patient survival [38]. Prolonging the treatment time boosts MSC effectiveness in end-stage liver disease and enhances the aforementioned signs and symptoms [39]. To sum up, it is necessary to define the ideal time for achieving favorable efficacy during MSC infusion. The time subgroup analysis of the MELD score with a random-effects model indicated that MSC infusion reduced the MELD score in comparison to the control group after 1, 6, and 9 months. However, after two weeks and a year, there was no statistically significant difference in MELD score between the MSC group and the control group. Similarly, ALB levels of the MSC group were significantly improved at 2 weeks, 1 month, 3 months, and 6 months after infusion. Reciprocally, the differences in ALB levels were statistically undistinguishable at 1 week and 12 months after MSC infusion. Therefore, these differences indicated that 6 months or 9 months after the infusion is an important time point for re-infusion of MSCs if the frequency of infusion is taken into account in future clinical studies. This might contribute to ensuring the efficacy and preservation of liver function.

Among Cochrane-registered(www.cochranelibrary.com) clinical trials of MSC treatment for LC, the majority of infused cell types are BM-MSCs and UC-MSCs. Presently, the MSCs originated from bone marrow within 7 of the 11 enrolled studies. Typically, MSCs can be extracted from multiple human tissues, and the homing and differentiation ability varies among different types of MSCs [40, 41]. In other words, it is necessary to take cell types into account when selecting a course of MSC treatment. Unfortunately, the small sample size within the current analysis could not meet the requirement for conducting a subgroup analysis of the effectiveness concerning the cell types. Even though previous meta-analyses demonstrated that BM-MSCs had superior therapeutic effects than UC-MSCs [42], it is still an unaddressed problem that which kind of MSCs exert the best therapeutic efficacy in LC. Existing studies have documented that human menstrual blood stem cells are capable of developing into functional stem cell-like cells [43]. In addition, human menstrual blood stem cells ameliorated liver fibrosis progression in animal models [44]. Moreover, one of the currently included studies examined the therapeutic outcomes of patients infused with differentiated and undifferentiated MSCs, respectively. These collectively offer novel perspectives for the advancement of different MSC treatments. However, it still calls for conducting more clinical trials to further determine the effectiveness of multiple kinds of MSCs in LC therapy in the future.

Excitingly, recent research has shed light on MSC-derived exosomes in the treatment of chronic liver disease. Similar to parental MSC activity, MSC-derived exosomes [45, 46] function as a mediator of intercellular communication between MSC and injured organ sites, which exerts effectiveness in various animal models of liver disease including acute liver damage, hepatic fibrosis, and hepatocellular carcinoma [47–50]. Typically, Exosomes are easier to obtain and store, and inherited with qualities like high reparability and low immunogenicity [51–53]. In light of this, MSC-derived exosomes are a promising alternative strategy for the treatment of LC.

Currently, different transplantation methods have raised questions about the influence on the efficacy of MSC treatment in LC. The four main transplantation routes are as follows: the hepatic artery route has a high rate of MSC colonization and survival, while the portal vein route is complicated and vulnerable to severe bleeding and vascular embolism aroused by portal hypertension in patients. The peripheral vein route is straightforward, less harmful, and easily repeated. The intraperitoneal route is vulnerable to abdominal infection and adhesion, thus influencing the migration of MSC cells [54–56]. According to studies based on animal models, BMSC transplantation through the portal and tail veins achieved similar improvements in liver function in LC rats [57]. Herein, the route of MSC transplantation should be taken into consideration as well. Hepatic artery and vein are preferred infusion routes in 10 enrolled clinical studies. As follows, we conducted infusion route subgroup analysis of ALB levels and MELD score, respectively. The results showed that the hepatic artery subgroup exerts increased ALB levels and reduced the MELD score. On the contrary, no statistically significant differences were observed in the intravenous subgroup. This could be attributed to that hepatic artery injections favor MSC's homing process during treatment. However, hepatic arterial injection also faces clinical limitations, such as high surgical risk and poor patient experience. Furtherly, this conclusion should be interpreted cautiously and more research is needed to search for an optimized infusion route in LC treatment.

MSCs are capable of directly differentiating into tissue cells in a particular microenvironment, thus replacing the injured cells [58]. Meanwhile, the fate of MSCs is regulated by a wide range of instructive signals from the microenvironment, which consists of many biomolecules (soluble and insoluble) and biomechanical forces [59]. The homing and migration of MSCs are affected by a variety of factors such as cell number and route of administration [60]. Unfortunately, no standard cellular dosage regimen is currently available for clinical MSC treatment in LC. In this meta-analysis, the dose subgroup analyses showed that the MSC group exerted significantly increased ALB levels in low-dose (1 × 10^6^ cells/kg) (SMD = 1.68; 95% CI [1.10, 2.25]; *p* < 0.00001) subgroup but not in the high-dose (1.0 × 10^7^–3.9 × 10^7^ cells/kg) subgroup in comparison with the control group. This suggested that a higher cell dose might not achieve the desired therapeutic effect. It was previously reported that the efficacy of high-dose MSC infusion was compromised by cell infusion complications such as portal hypertension and cellular embolism [61]. To achieve favorable therapeutic results, the ideal cell dose range should be determined in detail according to the patient's weight, clinical condition, route of administration, and cell type [8]. Therefore, clinical double-blind randomized controlled trials with more graded cell doses need to be carried out in the future.

As for secondary indicators, such as ALT, AST, TBIL, and INR biochemical markers which serve as a guide for clinical management of liver function status [62]. Whereas, no statistically significant difference was detected between the MSC group and the control group in our temporal subgroup analysis of AST, ALT, and INR. This might be due to the disease's etiology, type, small patient population, age and sex ratios of patients, and inconsistent follow-up duration between studies [63].

Upon determining MSC effectiveness, the effects of MSC on LC complications, HCC incidence, and mortality rates should also be analyzed. According to descriptive analysis results, MSC substantially relieves portal hypertension in LC patients and subsequently decreases ascites [64, 65]. Besides, our meta-analysis results showed that MSC transplantation decreased mortality and incidence of HCC in patients with LC. Typically, it is indicated that MSC impedes the development of HCC by modulating the immune state and microenvironment [66]. However, there are few clinical trials focused on the long-term prognosis of MSC in the treatment of LC, thus a large number of clinical RCTs are urgently required.

In this meta-analysis, we assessed the efficacy and safety of MSC in the therapy of LC and offered suggestions for clinical application. Our meta-analysis still has certain limitations. On the one hand, we assessed and examined the heterogeneity of enrolled studies and discovered a high degree of heterogeneity in ALB levels. The study performed by Fang et al. in which stem cells were extracted from the UC, might be the cause of heterogeneity [22]. On the other hand, the subgroup analysis was carried out to identify the potential factors that might affect MSC in the therapy of LC (such as time, cell type, route of infusion, and etiology). Even though the subgroup analysis and descriptive analysis produced preliminary findings, conclusions that MSC infusion reduces MELD score, increases ALB levels and improves LC complications might be biased. Consequently, more rigorous evaluations of MSC treatment in LC are required in the future. Last but not least, the pooled results could be skewed because there were only eleven retrospective studies. Therefore, further multicenter, large sample, long follow-up randomized controlled trials need to be carried out to address pressing concerns such as the suitable re-infusion time point, route, and quantity of cells in MSC transplantation therapy.

## Conclusion

In conclusion, MSC is safe and effective for treating LC. However, it is urgent to establish a standard treatment protocol to fully maximize the potential of MSC, which involves the optimization of re-infusion time point, route, frequency of infusion, and dose of cells. Collectively, these may facilitate further understanding of MSC treatment for LC and its pathophysiology, thus further improving the therapeutic effects. Besides, exosomes from MSCs are also expected to be clinically utilized for the treatment of LC. In the future, MSC and MSC-derived exosomes could be new strategies for the treatment of LC.

### Supplementary Information

**Additional file 1. Figure S1:** Forest plot of secondary indicators: **(A):** ALT levels **(B):** AST levels. **(C):** TBIL levels. **(D):** INR levels. **Figure S2:** Time subgroup of ALT levels. **Figure S3:** Time subgroup of AST levels. **Figure S4:** Time subgroup of TBIL levels.

**Additional file 2**. Detailed search strategy.

**Additional file 3**. PRISMA 2020 Checklist.

## Data Availability

Additional data used to support this study are presented in the Supplementary Material, further inquiries can be directed to the corresponding author.

## Abbreviations

MSCs: Mesenchymal stem cells
MELD: Model of end-stage liver disease
BM-MSCs: Bone marrow-derived MSCs
UC-MSCs: Umbilical cord-derived MSCs
LC: Liver cirrhosis
HCC: Hepatocellular carcinoma
PIIICP: Procollagen III C terminal peptide
PIIINP: Procollagen III N-terminal peptide
ALB: Albumin
TBIL: Total bilirubin
INR: International normalized ratio
ALT: Alanine aminotransferase
AST: Aspartate aminotransferase
RCTs: Randomized controlled trials
HBV: Hepatitis B virus
HCV: Hepatitis C virus
AC: Alcohol consumption
AE: Adverse event
