# Supplementary material for: Efficacy and safety of mesenchymal stem cell therapy in liver cirrhosis: a systematic review and meta-analysis
Source: Stem Cell Res Ther. 2023 Oct 20;14:301. doi: 10.1186/s13287-023-03518-x (PMC10590028; doi:10.1186/s13287-023-03518-x)
Supplement: Supplementary file 1 — Additional file 1. Figure S1: Forest plot of secondary indicators: (A): ALT levels (B): AST levels. (C): TBIL levels. (D): INR levels. Figure S2: Time subgroup of ALT levels. Figure S3: Time subgroup of AST levels. Figure S4: Time subgroup of TBIL levels. [file 13287_2023_3518_MOESM1_ESM.docx]

**Supplementary Materials**

**A**

**
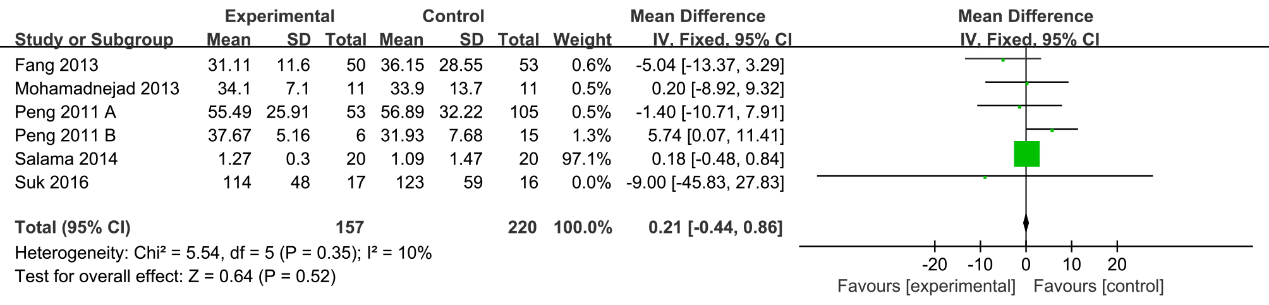
**

**B**


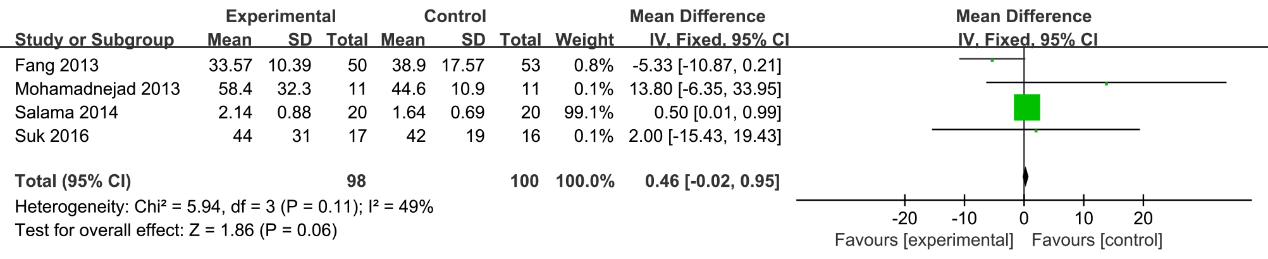


**C**


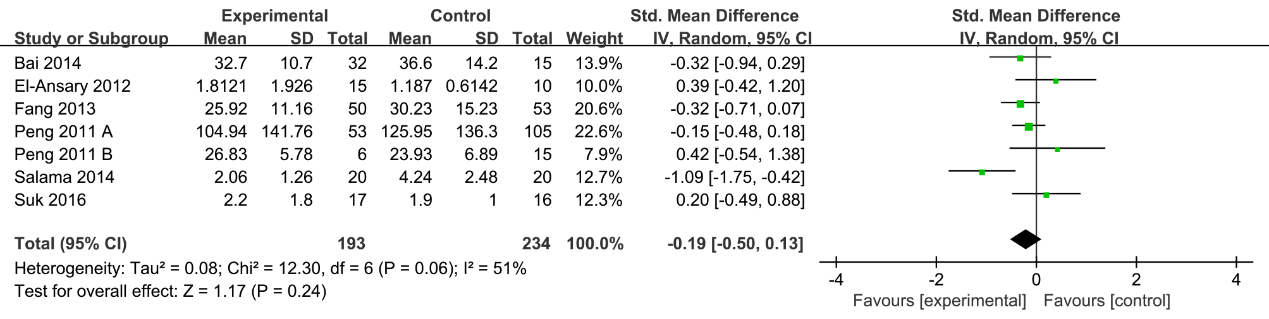


**D**


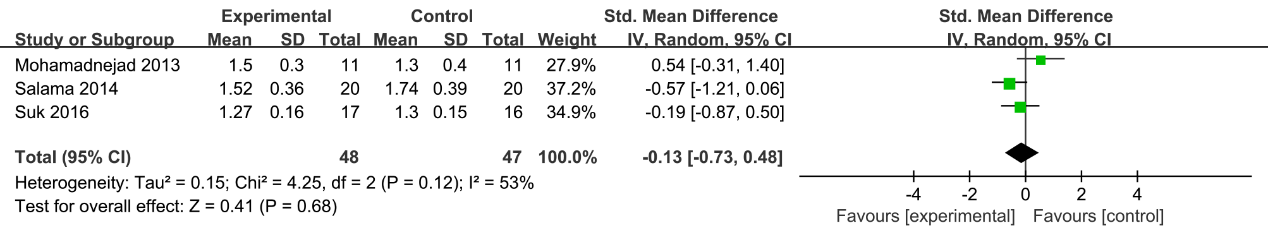


**Figure S1:** Forest plot of secondary indicators: (A): ALT levels (B): AST levels. (C): TBIL levels. (D): INR levels.


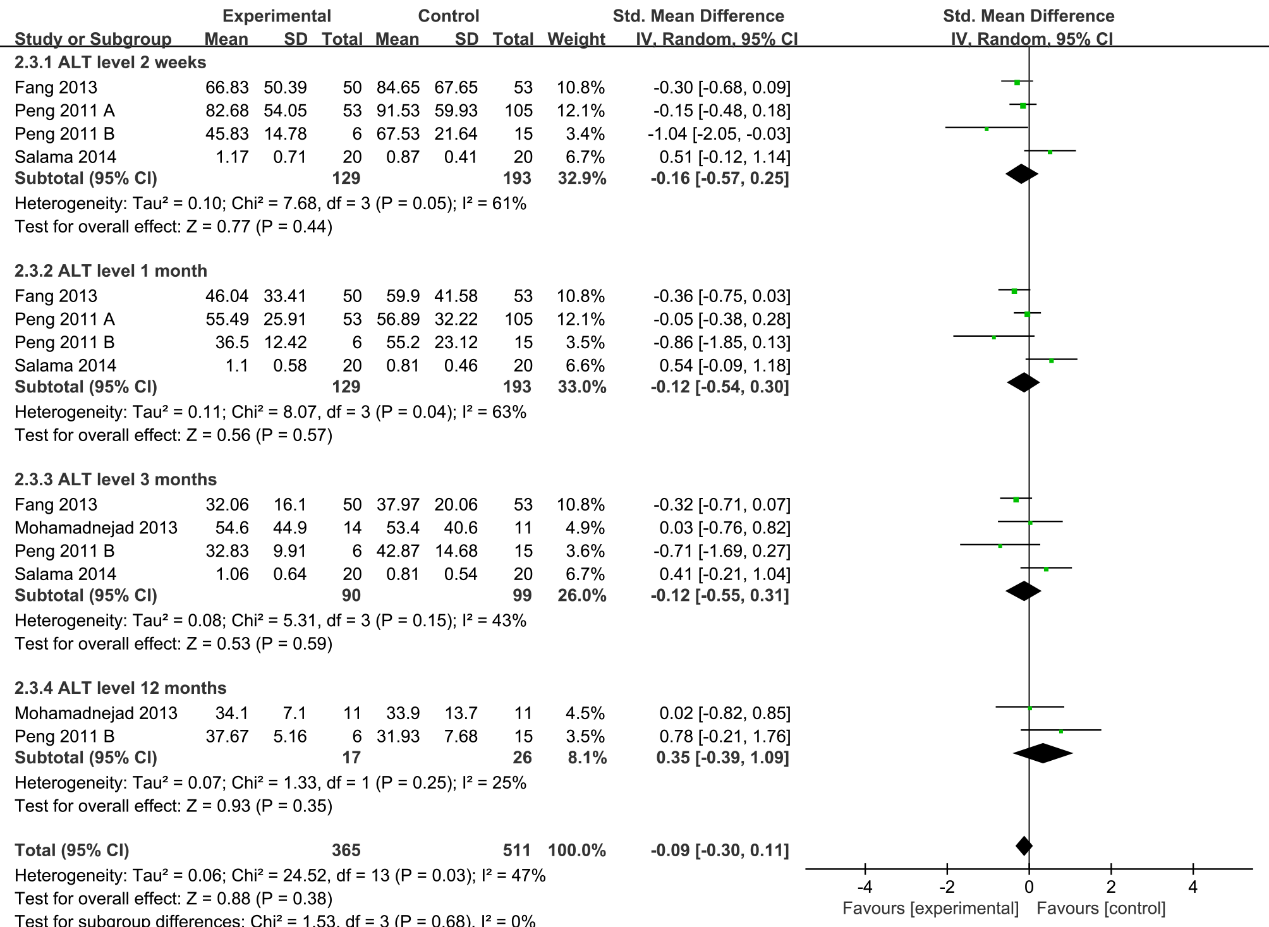


**Figure S2:** Time subgroup of ALT levels.

**
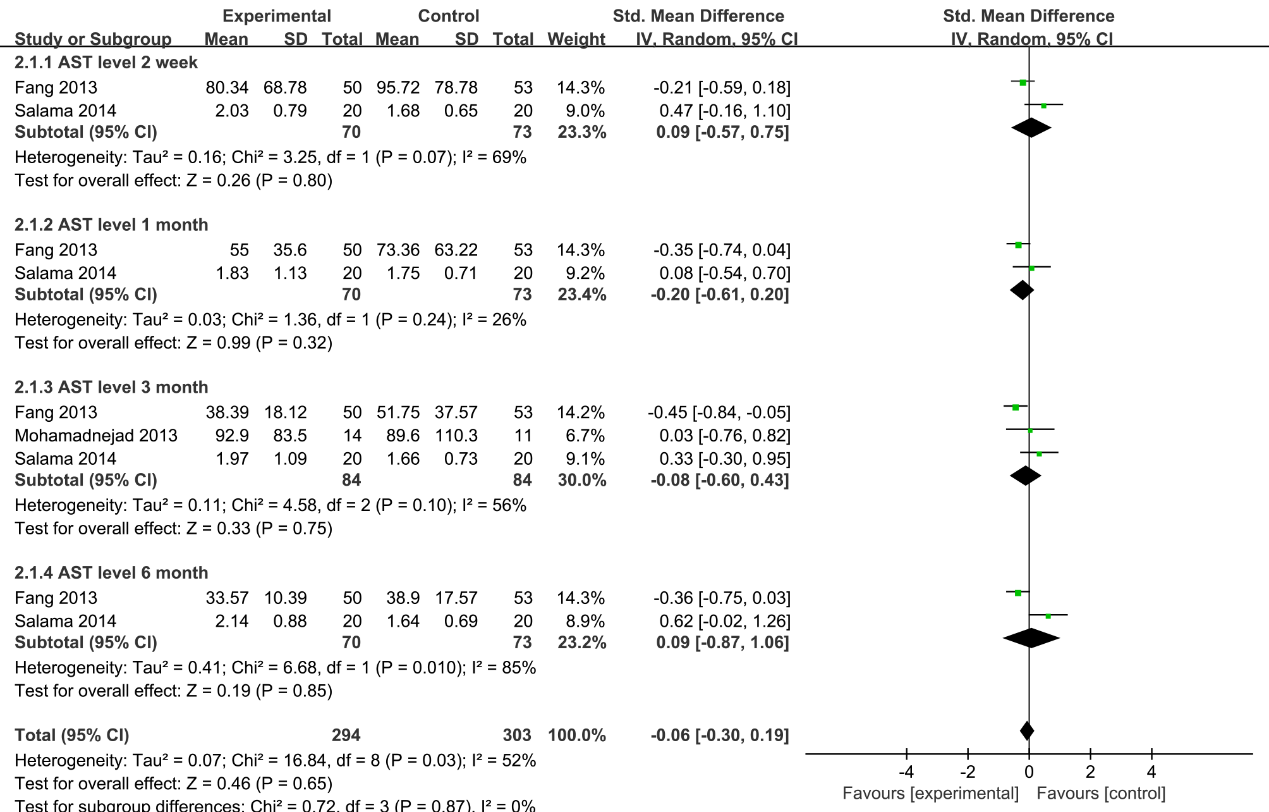
**

**Figure S3:** Time subgroup of AST levels.


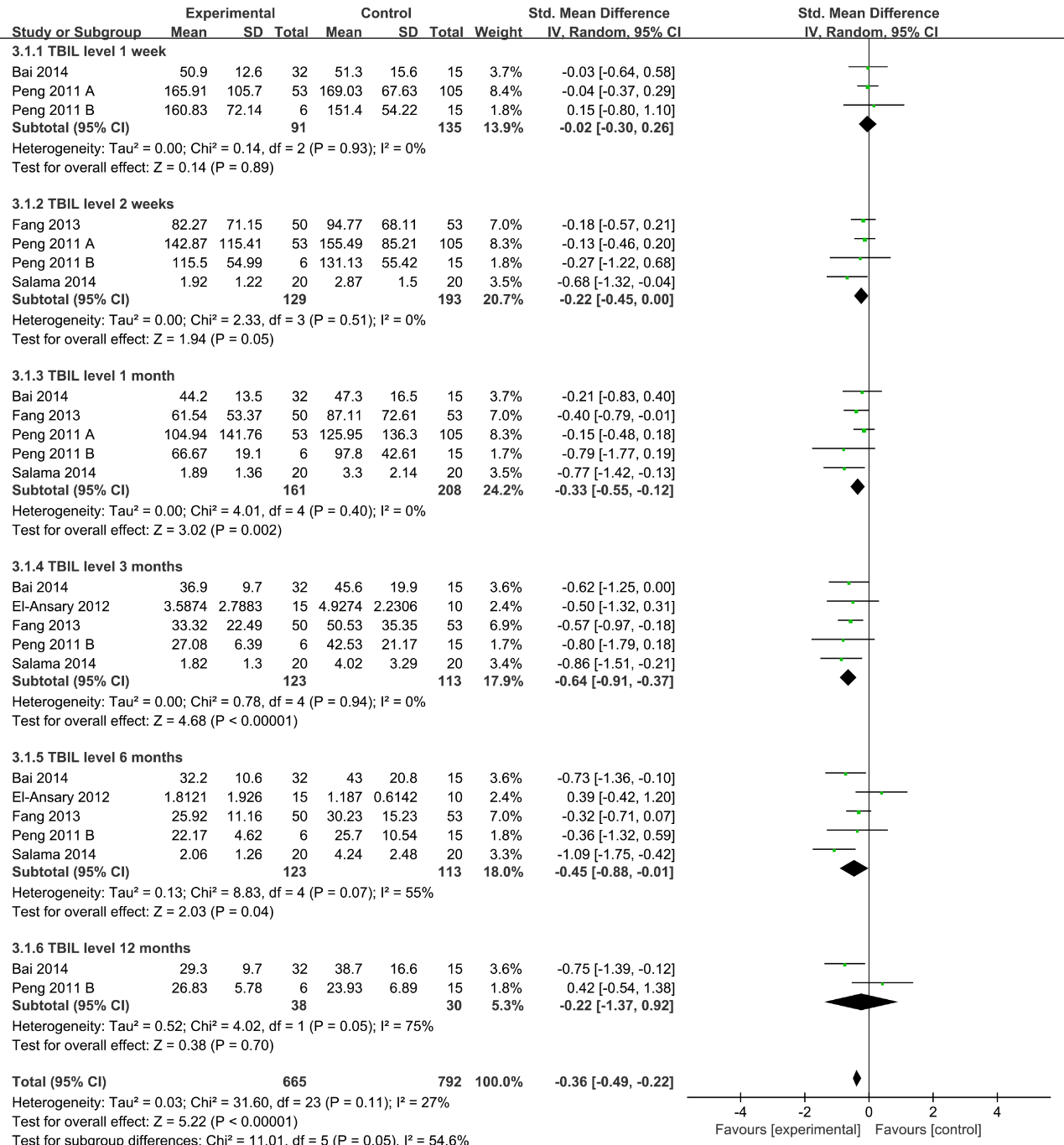


**Figure S4:** Time subgroup of TBIL levels.
